# Immediate Effect of Dry Needling at Myofascial Trigger Point on Hand Spasticity in Chronic Post-stroke Patients: A Multicenter Randomized Controlled Trial

**DOI:** 10.3389/fneur.2021.745618

**Published:** 2021-10-29

**Authors:** Zengqiao Zhang, Wu Wang, Yongjia Song, Tianjun Zhai, Yan Zhu, Liming Jiang, Qunfeng Li, Lei Jin, Kunpeng Li, Wei Feng

**Affiliations:** ^1^Department of Rehabilitation, Seventh People's Hospital of Shanghai University of Traditional Chinese Medicine, Shanghai, China; ^2^School of Rehabilitation Science, Shanghai University of Traditional Chinese Medicine, Shanghai, China; ^3^Department of Neurological Rehabilitation, Shanghai Second Rehabilitation Hospital, Shanghai, China; ^4^Department of Rehabilitation, Shanghai Hudong Hospital, Shanghai, China

**Keywords:** stroke, hand spasticity, dry needling, myofascial trigger point, immediate effect

## Abstract

**Background:** Hand spasticity after stroke influences the rehabilitation of hand function. Immediate and effective relief of spasticity potentially creates conditions for later rehabilitation training, which has far-reaching significance in the smooth transition of patients to the recovery period.

**Objective:** To evaluate the immediate effect of dry needling (DN) at myofascial trigger point on hand spasticity in stroke patients.

**Methods:** This was a prospective, evaluator blind, multicenter, randomized controlled study. A total of 210 participants were randomly divided into DN group (DN, *N* = 70), sham dry needling group (SDN, *N* = 70), and control group (*N* = 70). Participants in the DN group were treated with DN at myofascial trigger point five times (30 min each time) every week for 4 weeks. Subjects in the SDN group were manipulated the same way as in the DN group, except that the acupuncture site was located in the area adjacent to the myofascial trigger point, which constituted a SDN. Routine rehabilitation treatment was performed for participants in the two groups and in the control group. The primary evaluation index was the immediate effect of hand spasticity relief. Secondary evaluation indicators included the cumulative effect of hand spasticity relief from baseline to week 4, and the changes in flexion angles of the wrist, thumb, and fingers 2–5 in the rest position before, immediately after, and 4 weeks after intervention.

**Results:** The immediate effective rate of spasticity relief (thumb, fingers 2–5, and wrist) of patients with different degrees of spasticity in the DN group was higher than that in the control and SDN groups (thumb, χ^2^ = 55.833, *P* < 0.001; fingers 2–5, χ^2^ = 68.096, *P* < 0.001; wrist, χ^2^ = 49.180, *P* < 0.001) (*P* < 0.05). The effective rate of spasticity relief from baseline to 4 weeks in the DN group exceeded that in the control group and SDN groups (thumb, χ^2^ = 8.806, *P* = 0.012; fingers 2–5, χ^2^ = 8.087, *P* = 0.018; and wrist, χ^2^ = 8.653, *P* = 0.013) (*P* < 0.05). No difference in immediate and cumulative effect was found between the control group and SDN group. The change of joints flexion angles in resting position before and after each treatment in the DN group was higher than that in the control and SDN groups (*P* < 0.05), but it was not significantly different between the control group and SDN group. At 4 weeks, although the change in the DN group was higher than that in the control group and SDN group, this difference was not statistically significant (*P* > 0.05).

**Conclusion:** Dry needling can relieve varying degrees of hand spasticity instantly in post-stroke.

**Trial Registration:** www.chictr.org.cn, ChiCTR1900022379.

## Introduction

Stroke is a common disease that has seen an unprecedented rise in incidence, disability, and mortality (1). In spite of the declining mortality of stroke over the years, the disability rate is still soaring (2). The predominant association between physical disability and muscle spasticity delay the recovery time of limb function of patients (3). The incidence of spasticity can reach 42.6% in the chronic phase (>3 months post-stroke) (4). And there are different strategies by which spasticity can obstruct the improvement of hand function. As such, treating spasticity is a crucial approach to post-stroke management. In most cases, management of hypertonia occupies the majority of each rehabilitation session, and this reduces therapeutic efficiency greatly. This process is so distressing that patients consider discontinuing treatment, underscoring the need for immediate spasm relief, which is a prerequisite for rehabilitation of hand function with subsequent therapeutic approaches.

Conventional therapies for post-stroke spasticity primarily comprise physical therapy, surgical intervention, and pharmacotherapy (5). Physical therapy has demonstrated promising results in the management of post-stroke patients with limb spasticity. However, corresponding standards or clinical guidelines to guide the implementation of physical therapy, such as frequency or duration, are lacking. In addition, the majority of physical therapy interventions take a long duration, resulting in poor patient compliance and greatly reduced treatment efficacy (5). Although pharmacotherapy exerts some beneficial effects in post-stroke patients with limb spasticity, prolonged use of anti-spasmodics poses a risk of adverse effects and drug resistance; also, the high cost limits their application (6, 7). Surgical intervention can be a reliable option for post-stroke patients with severe spasticity; however, surgical treatment currently has few clinical applications because it is associated with high risk, numerous complications, and inaccurate effects (8). Therefore, a facile, cost-effective, and effective method is needed to offer immediate spasm relief and set the stage for rehabilitation training.

The DN technique was first put forward by Janet Travell in the 1940s. The American Physical Therapy Association (APTA) defined DN as a common intervention approach that entails the penetration of the skin with thin needles at the myofascial trigger point, muscle, and connective tissue to relieve musculoskeletal pain and dyskinesia (9). In recent years, mounting evidence supports a role for dry acupuncture at myofascial trigger points in improving dystonia in patients with neurological diseases (10). Although DN had a positive effect on lower limb spasticity in stroke patients, it had a negative effect on upper limb spasticity (11, 12). However, these results are derived from studies with low quality. Therefore, further high-quality studies are needed to confirm or refute the effect of DN on spasticity at the trigger point.

In our previous clinical practice, we found that the myofascial trigger point was repeatedly touched between the first metacarpal bone and the second metacarpal bone in post-stroke patients with hand spasticity. Dry needling (DN) at this myofascial trigger point elicited immediate relief from spasm, but a robust randomized controlled trial to provide further supportive evidence was lacking. In the present work, we performed a multicenter, prospective, randomized controlled trial to further evaluate the immediate efficacy of DN at myofascial trigger point in post-stroke patients with hand spasticity.

## Materials and Methods

### Study Design

This prospective, multicenter, three-arm, randomized controlled clinical trial was performed in line with the criteria of relevant trial guidelines and was approved by the Institutional Review Board and the Ethical Committee (2018-IRBQYYS). We registered the clinical trial on the Chinese clinical trial registry (ChiCTR1900022379) before the enrolment of the first participant.

### Participants

Participants were recruited from the Seventh People's Hospital Affiliated with the Shanghai University of traditional Chinese medicine, Shanghai Second rehabilitation hospital, and Shanghai Hudong hospital through the web platform, outpatient, and inpatient clinical poster advertisements.

### The Inclusion Criteria Were as Follows

① Clinically diagnosed with stroke (13); ② Brunnstrom stages ranged from II to IV; ③ spasticity of the hand [Modified Ashworth Scale (MAS) score 1^+^-3); ④ aged between 50 and 70 years; ⑤ could understand the content of the scale and cooperate with the evaluation and treatment; ⑥ agreed to engage in the trial and signed the informed consent.

### The Exclusion Criteria Were as Follows

① Secondary Parkinson's disease; ② aphasia, conscious, or cognitive impairment; ③ severe bleeding tendency or infection of treatment site; ④ received other related treatment in the past 3 months; ⑤ other causes of hand spasticity; ⑥ combined with muscle contracture or joint deformity; ⑦ pregnant and lactating women; ⑧ fear of acupuncture or fainting.

Researchers in our team selected the qualified participants according to the relevant standards. Eligible participants were informed of the research plan and signed informed consent after preliminary screening were further assessed by a therapist.

### Randomization and Masking

Eligible participants were allocated in a 1:1:1 ratio. Nine randomized groups were stratified in three hospitals. The random sequence was performed using a computer-generated code generated using SAS software version 9.4 (SAS Institute) with a random seed of 2,118 generated centrally at the Clinical Trials Center of Shanghai Seventh People's hospital. The generated random sequence was kept by a specially designated person. The clinical research coordinator recorded the information of participants, obtained random numbers, and determined their allocation. The research assistant screened and recruited participants and assigned them random numbers in the entire research process. The evaluator of the results was responsible for recording the scale data. All rehabilitation therapists, outcome assessors, and statisticians were blind to group assignment.

### Study Interventions

All interventions were performed by practitioners with relevant legal qualifications and extensive clinical experience.

#### Dry Needling Group

Participants in this group were treated with DN at myofascial trigger point five times a week (30 min each time) for 4 weeks. In addition, they were given routine rehabilitation therapy at the same frequency and intensity as the control group.

**Figure 1:**
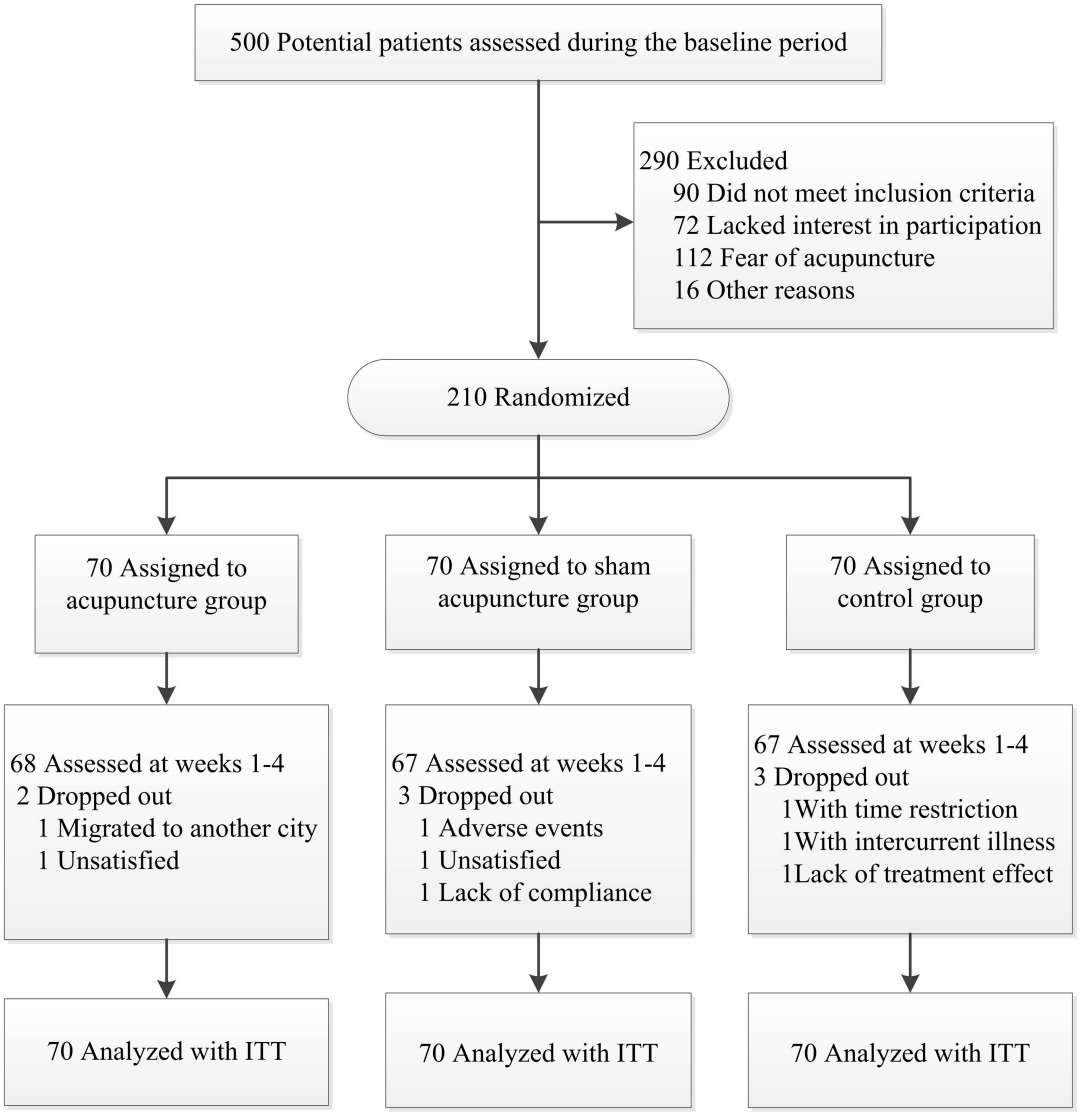
Flowchart of the screening, enrollment, and randomization.

*The location of myofascial trigger point:* The patient remained seated or supine, with the doctor standing on the patient's side. Briefly, the operator's thumb and the area between the first metacarpal bone and the second metacarpal bone on the back of the patient's hand were disinfected with a 75% alcohol cotton ball. Subsequently, the operator applied unidirectional pressure from the distal end to the proximal end in the area between the first metacarpal bone and the second metacarpal bone on the dorsal side of the patient's palm with the sterilized screw surface of the thumb. In this process, the operator touched the cord like nodules, and the patient would feel an obvious pain. Deep pressure led to distal referred pain, which is the myofascial trigger point (Figure 1).

*Method of needling:* After routine disinfection, the operator swiftly penetrated a sterile needle (0.3 mm ^*^ 25 mm) vertically into the myofascial trigger point. The success criteria of acupuncture were local pain, distal finger pain, and finger twitch. The needle was kept for 30 min following the induction of a convulsive reaction.

#### Sham Dry Needling Group

Participants in this group received sham dry acupuncture five times a week (30 min each time) for 4 weeks. The acupuncture needle was inserted 2 mm lateral to myofascial trigger point to a depth of 2 mm without manual stimulation (14). In addition, subjects received routine rehabilitation therapy at the same frequency and intensity as the control group.

#### Control Group

The participants in this group received routine rehabilitation treatment, including recumbent position, neurodevelopmental treatment, and activities of daily living (ADL) treatment, five times a week for 4 weeks. The participants were also given routine anti-stroke treatment.

### Outcome Measures

#### Primary Outcome Measurement

The primary outcome was the immediate effect of DN at myofascial trigger point on thumb spasticity relief. The spasticity grade of flexor muscles was assessed with MAS before and immediately after each treatment. Treatment was considered effective when MAS score was reduced by at least one grade.

#### Secondary Outcome Measures

Secondary outcomes included the immediate effect of DN at myofascial trigger point on fingers 2–5 and wrist spasticity relief, the cumulative effect of hand (thumb, fingers 2–5, and wrist) spasticity relief from baseline to week 4, and changes of the flexion angles of the wrist, thumb (metacarpophalangeal joint, interphalangeal joint), and fingers 2–5 (metacarpophalangeal joint, proximal interphalangeal joint, distal interphalangeal joint) of the affected hand before and right after each treatment and at the end of 4-week intervention. Participants were asked to adopt a natural position during the assessments. In this study, joint angle was measured at the condition that patients were in a supine position with their arms placed on both sides of body, elbow stretched, and palm up. Then fix the protractor so that its axis was aligned with the center of the joint. In addition, subgroup analysis was performed to provide further evidence for our findings.

### Statistical Analyses

The purpose of this study was to explore whether there was any difference in the effective rate of immediate relief in post-stroke patients with hand spasticity among the DN group, the sham dry needling (SDN) group, and the control group. According to our preliminary study, the effective rates of immediate remission of thumb spasm after each treatment were assumed to be 55% (control group), 80% (DN group), and 50% (SDN group), respectively. Herein, assuming a dropout rate of 20%, the allowable error of 5%, and the test power of 90%, we needed a sample size of 210 (70 in each group).

Descriptive statistics were performed on the baseline characteristics of patients. Student *t*-test or one-way ANOVA were applied for those who fit a normal distribution to continuous variables, whereas Kruskal–Wallis *H*-test was used for others. For categorical variables, the chi-square test or Fisher exact test were used. *P*-value <0.05 denoted statistical significance. Notably, to avoid type I error when performing multiple comparisons, adjusted *P*-value was calculated as *P*-value of 0.05 divided by the number of comparisons.

## Results

### Study Participants

**Figure 2:**
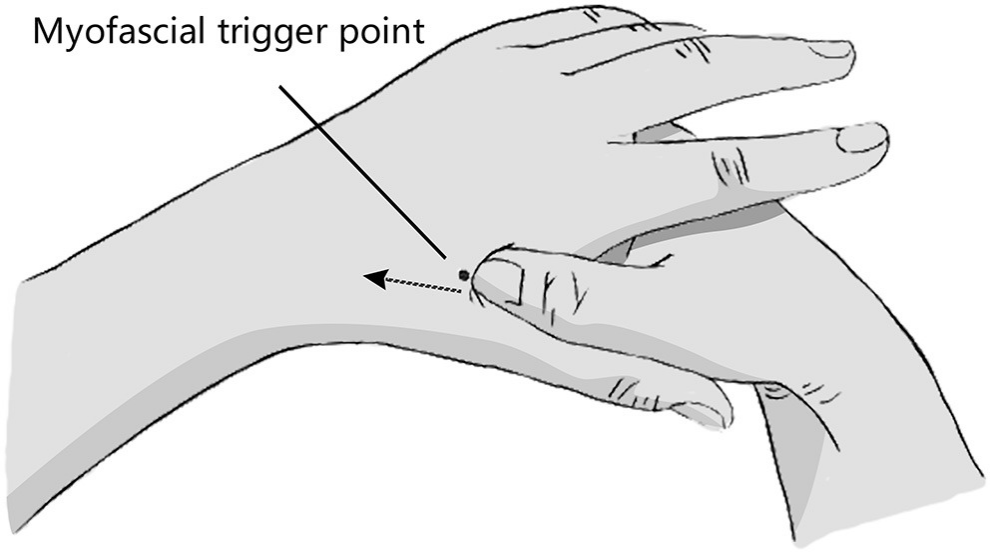
The location of myofascial trigger point.

After screening 500 subjects, 290 were eligible for inclusion, but we ended up enrolling 210 participants since some did not meet inclusion criteria (90 patients) or lacked interest in participation (72 patients), feared acupuncture (112 patients), or other reasons (16 patients). Figure 2 present a flow chart of patient screening, enrollment, and randomization. Baseline characteristics of the randomized patients were well-balanced in the three treatment groups (Table 1). There was no significant difference in baseline characteristics among the three groups.

**Table 1:** Baseline characteristics.

**Characteristics**	**DN group (*n* = 70)**	**SDN group (*n* = 70)**	**CON group (*n* = 70)**	**χ^2^**	***P***
**Age, mean (SD), years**	66.17 (9.84)	62.97 (11.53)	65.07 (8.50)	1.837	0.162
**Gender, No (%)**					
Female	23 (32.90)	26 (37.10)	22 (31.40)	0.553	0.758
Male	47 (67.10)	44 (62.90)	48 (68.60)		
Duration of illness, mean (SD), month	12.67 (3.09)	13.41 (2.98)	12.54 (3.04)	1.662	0.192
**MAS score^§^, No (%)**					
1+	37 (52.90)	31 (44.30)	36 (51.40)	1.378	0.848
2	26 (37.10)	29 (41.40)	26 (37.10)		
3	7 (10.00)	10 (14.30)	8 (11.40)		
**Types of stroke, No (%)**					
Cerebral ischemic stroke	52 (74.30)	46 (65.70)	47 (67.10)	1.381	0.501
Cerebral hemorrhagic stroke	18 (25.70)	24 (34.30)	23 (32.90)		
**Affected limb, No (%)**					
Left	34 (48.60)	33 (47.10)	37 (52.90)	0.495	0.781
Right	36 (51.40)	37 (52.90)	33 (47.10)		

*DN, dry needling; SDN, sham dry needling; CON, control*.

§ *MAS, Modified Ashworth Scale, The Modified Ashworth Scale is a measure of spasticity (muscle tone) in the paralyzed arm; scores range from 0 to 4 at each joints, with higher scores indicating more severe spasticity; P-value of <0.05 was considered significant*.

### Primary Outcome

According to the MAS, the effective rate of thumb spasticity relief from baseline to after each treatment in the DN group was higher than that in control and SDN groups (χ^2^ = 55.833, *P* < 0.001) (Table 2); however, no significant difference was reported between the control group and SDN group.

**Table 2:** Effective rate of thumb spasticity relief from baseline to after each treatment.

**Group**	***N***	**Effective cases**	**Effective rate (%)^γ^**	**χ^2^**	***P*-value**
**DN**	**70**	67	95.71	55.833	<0.001
**SDN**	**70**	30	42.86^※^		
**CON**	**70**	29	41.43^※^^#^		

*N, number of cases included in each group; DN, dry needling; SDN, sham dry needling; CON, control*.

γ *Bonferroni correction was used for multiple comparisons between groups, α= 0.05/3 = 0.017*.

※ * Statistically significant difference from DN group*.

# *No statistical significance as compared to SDN group; P-value of <0.05 was considered significant*.

### Secondary Outcomes

#### Effective Rate of Spasticity Relief From Baseline to After Each Treatment in Fingers 2–5 and Wrist

According to the MAS, the effective rate of fingers 2–5 and wrist spasticity relief from baseline to after each treatment in the DN group was higher than that in control and SDN groups (fingers 2–5, χ^2^ = 68.096, *P* < 0.001; wrist, χ^2^ = 49.180, *P* < 0.001) (Table 3); however, no significant difference was reported between the control group and SDN group.

**Table 3:** Effective rate of spasticity relief from baseline to after each treatment in fingers 2–5 and wrist.

**Sites**	**Group**	***N***	**Effective cases**	**Effective rate (%)^γ^**	**χ^**2**^**	***P*-value**
Fingers 2–5 flexor spasticity	DN	70	69	98.57	68.096	<0.001
	SDN	70	29	41.43^※^		
	CON	70	26	37.14^※^^#^		
Wrist flexor spasticity	DN	70	64	91.43	49.180	<0.001
	SDN	70	28	40.00^※^		
	CON	70	29	41.43^※^^#^		

*N, number of cases included in each group; DN, dry needling; SDN, sham dry needling; CON, control*.

γ *Bonferroni correction was used for multiple comparisons between groups, α= 0.05/3 = 0.017*.

※ *Statistically significant difference from DN group*.

# *No statistical significance as compared to SDN group. P-value of <0.05 was considered significant*.

#### Subgroup Analysis of the Immediate Effective Rate of Different Degrees of Hand Spasticity

The immediate effective rate of thumb spasticity relief (Table 4) of patients with different degrees of spasticity in the DN group was higher than that in the control and SDN groups (1+, χ^2^ = 8.682, *P* = 0.013; 2, χ^2^ = 35.533, *P* < 0.001; 3, *P* = 0.001), however, no significant difference was reported between the control group and SDN group. This tendency was also seen in fingers 2–5 (Table 5) and wrist (Table 6).

**Table 4:** Immediate effective rate of thumb spasticity relief in patients with different degree of spasticity.

**MAS score^§^**	**Group**	***N***	**Effective cases**	**Effective rate (%)^γ^**	**χ^**2**^**	***P*-value**
1+	DN	37	36	97.30	8.682	0.013
	SDN	31	24	77.42^※^		
	CON	36	26	72.22^※^^#^		
2	DN	26	20	76.92	35.533	<0.001
	SDN	29	4	13.79^※^		
	CON	26	2	7.69^※^^#^		
3	DN	7	6	85.71	NA	0.001^η^
	SDN	10	1	10.00^※^		
	CON	8	1	12.50^※^^#^		

*N, number of cases included in each group; DN, dry needling; SDN, sham dry needling; CON, control*.

§ *>MAS, Modified Ashworth Scale, The Modified Ashworth Scale is a measure of spasticity (muscle tone) in the paralyzed arm; scores range from 0 to 4 at each joints, with higher scores indicating more severe spasticity*.

γ *>Bonferroni correction was used for multiple comparisons between groups, α = 0.05/3 = 0.017*.

※ *> Statistically significant difference from DN group*.

# *>No statistical significance as compared to SDN group; P-value of <0.05 was considered significant*.

η *>Fisher's exact test*.

*NA, Not Available*.

**Table 5:** Immediate effective rate of fingers 2–5 spasticity relief in patients with different degree of spasticity.

**MAS score^§^**	**Group**	***N***	**Effective cases**	**Effective rate (%)^γ^**	**χ^**2**^**	***P*-value**
1+	DN	37	37	100.00	20.342	<0.001
	SDN	31	22	70.97^※^		
	CON	36	20	55.56^※^^#^		
2	DN	26	22	84.62	32.625	<0.001
	SDN	29	5	17.24^※^		
	CON	26	5	19.23^※^^#^		
3	DN	7	7	100.00	NA	<0.001^η^
	SDN	10	2	20.00^※^		
	CON	8	1	12.50^※^^#^		

*N, number of cases included in each group; DN, dry needling; SDN, sham dry needling; CON, control*.

§ *MAS, Modified Ashworth Scale, The Modified Ashworth Scale is a measure of spasticity (muscle tone) in the paralyzed arm; scores range from 0 to 4 at each joints, with higher scores indicating more severe spasticity*.

γ *Bonferroni correction was used for multiple comparisons between groups, α = 0.05/3 = 0.017*.

※ *Statistically significant difference from DN group*.

# *No statistical significance as compared to SDN group*.

*P-value of <0.05 was considered significant*.

η *Fisher's exact test*.

*NA, Not Available*.

**Table 6:** Immediate effective rate of wrist spasticity relief in patients with different degree of spasticity.

**MAS score^§^**	**Group**	***N***	**Effective cases**	**Effective rate (%)^γ^**	**χ^2^**	***P*-value**
1+	DN	37	35	94.59	10.114	0.006
	SDN	31	21	67.74^※^		
	CON	36	24	66.67^※^^#^		
2	DN	26	17	65.38	16.09	<0.001
	SDN	29	6	20.69^※^		
	CON	26	5	19.23^※^^#^		
3	DN	7	5	71.43	NA	0.004^η^
	SDN	10	1	10.00^※^		
	CON	8	0	0.00^※^^#^		

*N, number of cases included in each group; DN, dry needling; SDN, sham dry needling; CON, control*.

§ *MAS, Modified Ashworth Scale, The Modified Ashworth Scale is a measure of spasticity (muscle tone) in the paralyzed arm; scores range from 0 to 4 at each joints, with higher scores indicating more severe spasticity*.

γ *Bonferroni correction was used for multiple comparisons between groups, α = 0.05/3 = 0.017*.

※ *Statistically significant difference from DN group*.

# *No statistical significance as compared to SDN group; P-value of <0.05 was considered significant*.

η *Fisher's exact test*.

*NA, Not Available*.

#### The Effective Rate of Spasticity Relief From Baseline to Four Weeks

According to the MAS, the effective rate of spasticity relief from baseline to 4 weeks in the DN group was higher than that in control and SDN groups (thumb, χ^2^ = 8.806, *P* = 0.012; fingers 2–5, χ^2^ = 8.087, *P* = 0.018; and wrist, χ^2^ = 8.653, *P* = 0.013) (Table 7); however, no significant difference was found between the control group and SDN group.

**Table 7:** Effective rate of spasticity relief from baseline to week 4.

**Sites**	**Group**	***N***	**Effective cases^γ^**	**Effective rate(%)^≠^**	**χ^**2**^**	***P*-value**
Thumb flexor spasticity	DN	70	55	78.57	8.806	0.012
	SDN	70	41	58.57^※^		
	CON	70	40	57.14^※^^#^		
Fingers 2–5 flexor spasticity	DN	70	57	81.43	8.087	0.018
	SDN	70	43	61.43^※^		
	CON	70	44	62.86^※^^#^		
Wrist flexor spasticity	DN	70	54	77.14	8.653	0.013
	SDN	70	39	55.71^※^		
	CON	70	40	57.14^※^^#^		

*N, number of cases included in each group; DN, dry needling; SDN, sham dry needling; CON, control*.

≠ *Intention-to-Treat Analysis, 2 dropped out in DN group, 3 dropped out in SDN group, 3 dropped out in control group*.

γ *Bonferroni correction was used for multiple comparisons between groups, α = 0.05/3 = 0.017*.

※ * Statistically significant difference from DN group*.

# *No statistical significance as compared to SDN group; P-value of <0.05 was considered significant*.

#### Changes of Joint Angles of Hand in Rest Position

Mean rank of joint angle change in hand rest position from baseline to after each treatment in the DN group were 148.97, 163.59, 160.28, 170.07, 169.27, and 169.91 at the wrist, MCP and IP of the thumb, MCP, DIP, and the PIP of the fingers 2–5, respectively; the corresponding changes in the control group were 85.38, 76.44, 78.53, 72.20, 72.34, 73.06 and 82.15, 76.48, 77.69, 74.23, 74.89, 73.53 in the SDN group. There was statistical significance in the DN group compared to the control group and SDN group, while *P* > 0.05 for comparisons between the control group and SDN group (Table 8). At 4 weeks, although the mean rank of the DN group was higher than that of the control group and SDN group, the difference was not statistically significant (*P* > 0.05), except for the comparison between the DN group and SDN group in the proximal interphalangeal joint of the fingers 2–5 (*P* = 0.026) (Table 9).

**Table 8:** Change of joint flexion angles in resting position before and after each treatment.

**Joint**	**Group**	**Median (*P*25, *P*75)**	**Mean rank^γ^**	**Kruskal–Wallis** ***H*****-test**	
				***H***	***P-*value**
Wrist	DN	31.50 (17.25, 40.25)	148.97	58.133	<0.001
	SDN	0.00 (0.00, 26.25)	82.15^※^		
	CON	0.00 (0.00, 27.25)	85.38^※^^#^		
MCP of thumb	DN	20.00 (15.00, 25.00)	163.59	101.389	<0.001
	SDN	0.00 (0.00, 9.25)	76.48^※^		
	CON	0.00 (0.00, 9.25)	76.44^※^^#^		
IP of thumb	DN	39.00 (30.00, 53.00)	160.28	90.427	<0.001
	SDN	0.00 (0.00, 30.00)	77.69^※^		
	CON	0.00 (0.00, 28.00)	78.53^※^^#^		
MCP of fingers 2–5	DN	22.00 (17.00, 28.00)	170.07	125.889	<0.001
	SDN	0.00 (0.00, 8.25)	74.23^※^		
	CON	0.00 (0.00, 7.25)	72.2^※^^#^		
PIP of fingers 2–5	DN	42.00 (36.00, 52.00)	169.27	122.796	<0.001
	SDN	0.00 (0.00, 26.25)	74.89^※^		
	CON	0.00 (0.00, 25.25)	72.34^※^^#^		
DIP of fingers 2–5	DN	35.00 (33.00, 51.00)	169.91	125.233	<0.001
	SDN	0.00 (0.00, 22.00)	73.53^※^		
	CON	0.00 (0.00, 24.00)	73.06^※^^#^		

*DN, dry needling; SDN, sham dry needling; CON, control; MCP, metacarpophalangeal joint; IP, interphalangeal joint; DIP, distal interphalangeal joint; PIP, proximal interphalangeal joint*.

γ *Bonferroni correction was used for multiple comparisons between groups, α = 0.05/3 = 0.017*.

※ * Statistically significant difference from DN group*.

# *No statistical significance as compared to SDN group; P-value of <0.05 was considered significant*.

**Table 9:** Change of joint flexion angles in resting position from baseline to week 4.

**Joint**	**Group**	**Median (*P*25, *P*75)**	**Mean rank^γ^**	**Kruskal–Wallis** ***H*****-test**	
				***H***	***P-*value**
Wrist	DN	14.50 (4.25, 30.00)	119.78^#^^ϕ^	6.108	0.047
	SDN	8.00 (0.00, 29.00)	98.67^ϕ^		
	CON	8.00 (0.00, 26.00)	98.05		
MCP of thumb	DN	10.00 (5.00, 14.00)	117.38	4.209	0.122
	SDN	8.50 (0.00, 12.00)	99.39		
	CON	6.00 (0.00, 13.00)	99.74		
IP of thumb	DN	26.50 (5.75, 30.00)	120.79^#^^ϕ^	6.965	0.031
	SDN	23.00 (0.00, 29.00)	98.06^ϕ^		
	CON	23.00 (0.00, 29.00)	97.65		
MCP of fingers 2–5	DN	10.00 (5.00, 13.00)	116.38	3.513	0.173
	SDN	8.00 (0.00, 14.00)	100.95		
	CON	8.00 (0.00, 13.00)	99.17		
PIP of fingers 2–5	DN	25.00 (17.75, 30.00)	121.84^ϕ^	8.004	0.018
	SDN	20.00 (0.00, 26.00)	95.28^※^		
	CON	20.00 (0.00, 28.00)	99.38^#^		
DIP of fingers 2–5	DN	24.50 (18.75, 26.25)	117.9	5.022	0.081
	SDN	21.00 (0.00, 25.25)	95.72		
	CON	22.00 (0.00, 27.00)	102.88		

*DN, dry needling; SDN, sham dry needling; CON, control; MCP, metacarpophalangeal joint; IP, interphalangeal joint; DIP, distal interphalangeal joint; PIP, proximal interphalangeal joint*.

γ *Bonferroni correction was used for multiple comparisons between groups, α = 0.05/3 = 0.017*.

※ * Statistically significant difference from DN group*.

# *No statistical significance as compared to SDN group*.

ϕ *No statistical significance as compared to CON group; P-value of <0.05 was considered significant*.

### Adverse Events

No serious adverse events requiring withdrawal were reported across the three groups (Table 10).

**Table 10:** Adverse events related to treatment.

**Adverse event^**a**^**	**Participant, No (%)**		
	**DN (*n* = 70)**	**SDN (*n* = 70)**	**CON (*n* = 70)**
Overall	1 (1.43)	2 (2.86)	0
Severe adverse events	0	0	0
Subcutaneous hematoma	1 (1.43)	1 (1.43)	0
Fainting	0	0	0
Sharp pain	0	1 (1.43)	0
Instrument fracture	0	0	0
Sticking of needle	0	0	0

*a: Adverse events were analyzed in all participants who received treatment. Adverse events were counted by type rather than frequency in the same participant. Adverse events with different types occurring in a single participant were defined as independent adverse events. An adverse event with multiple occurrences in a single participant was defined as 1 adverse event. DN, dry needling; SDN, sham dry needling; CON, control*.

## Discussion

The immediate efficacy of DN at the trigger point in the treatment of hand spasticity after stroke has been evaluated. In our clinical trial, DN could effectively relieve different degrees of hand spasticity in patients with stroke after each treatment. It also reduced the flexion angle of joints (wrist joint, thumb, and fingers 2–5) in the rest position of the hand. The results of this study showed that the effective rate of spasticity relief (immediate or 4 weeks) in the SDN group was equivalent to that in the control group. We speculated that this might be related to the distinctive location and manipulation of myofascial trigger points. Acupuncture at the non-myofascial trigger points was difficult to produce curative effect, excluding the placebo effect caused by acupuncture behavior itself. By discontinuing the intervention after 4 weeks, some level of cumulative effect was realized although it was not significant. Considering that the intervention cycle is too short, we speculate that it takes a certain time for the accumulation of stimulation effect before it reaches levels that produce best treatment effects. Because the curative effect of acupuncture is a gradual accumulation process, that is, the therapeutic effect improves gradually as the treatment course of acupuncture increases, this is termed as accumulation of post acupuncture effect. Based on previous research, we plan to increase the acupuncture and needle retention time, prolong the observation period, explore the time-effect relationship in acupuncture, longitudinally study the whole action cycle of acupuncture, and determine a more reasonable treatment scheme. DN treatment primarily reduces muscle tension to relieve limb spasms and create conditions for later rehabilitation training. These effects are beneficial to patients to develop in the direction of separation and coordination, and to allow their smooth transition to the recovery period.

Emerging evidence has implicated DN in the management of muscle spasticity in nervous system diseases, including spastic quadriplegia and post-stroke spasticity. The meta-analysis of Fernández-de-Las-Peñas et al. (11) found that the effect of DN on spasticity was mainly in the lower extremities, though the effect on related pain and motor function was inconclusive in the short-term follow-up. Elsewhere, Ghannadi et al. (15) found that deep DN exerts short-term effects by reducing the muscle spasm of stroke survivors and improving the lower limb function and gait. More evidence from Salom-Moreno's work showed that a single DN not only reduces the spasticity and extensive pressure sensitivity of patients with spasticity after stroke but also changes the plantar pressure by increasing the support surface and reducing the average pressure (16). Núñez-Cortés et al. (17) found that DN, either alone in combination with multimodal therapy, could effectively reduce stroke spasticity and improve passive range of motion in a short period. Echoing the above reports, Sánchez-Mila et al. (18) demonstrated that DN combined with Bobath therapy could effectively reduce spasticity, improve balance, range of motion, and maintain stable accuracy in stroke patients.

Although DN had a positive effect on lower limb spasticity of stroke patients, it had negative effects on upper limb spasticity. Mendigutia-Gómez et al. (12) revealed that DN, incorporated in the multimodal rehabilitation program, reduced the local pressure sensitivity and improved the range of motion of shoulder joint in stroke patients effectively; however, they reported no significant difference in muscle spasm relief. In another study, Cuenca Zaldívar et al. (19) found that addition of DN treatment to the standard physical therapy reduced the spasticity of the affected arm better but did not show additional effects in function, pain, and quality of life. More pieces of research by Ansari, Ghaffari, and Fakhari have shown that DN can reduce the upper limb spasm, improve the range of motion, and promote the recovery of joint function (20–22). In support of these findings, Lu et al. (23) demonstrated DN could immediately relieve finger flexor spasm, increase range of motion, and reduce motor unit action potential (MUAP), suggesting that there may be potential trigger points in spastic muscles of chronic stroke, which are related to spastic hypertension of flexor digitorum. These observations confirm our results that DN at the trigger point of the hand can effectively relieve hand spasticity after stroke. Additionally, Hernández-Ortiz et al. (24) found that the effect of DN on muscle tension (spasm) and upper limb function in stroke patients was not related to the intervention in and out of trigger points region. More research is warranted to further confirm or refute the effect of DN at the trigger point on muscle spasms.

Of note, the results presented here should be treated with caution because the majority of previous studies were scattered case reports or systematic reviews with low quality of data. In addition, the number of included cases was small and lacks the control group, and therefore were not eligible to prove the efficacy and safety of DN. It was not possible to explore the effect of DN alone because it was applied as a multimodal treatment. Contrary to previous studies, the present work used a larger sample size to conduct multicenter randomized controlled trials. The control group and sham acupuncture group were set up to exclude the effect of comfort treatment due to psychological factors, to better judge the curative effect of DN. Secondly, independent DN intervention and instant assessment of spasms were administered before routine rehabilitation training. This design is beneficial in the assessment of the effect of DN alone. In addition to the immediate effect, we also evaluated the cumulative effect and conducted a priori subgroup analysis (prior) to validate the internal consistency of clinical trial results and explore the most suitable beneficiary population. The results demonstrated that DN intervention at a single site myofascial trigger point could immediately relieve hand spasm after stroke. This approach is convenient and easy to promote.

Although the results of our multicenter randomized controlled trials are encouraging, potential limitations should be recognized. First, because we collected short-term results, whether the observed changes will last longer and whether the continuous stimulation given by the improvement of the needle can continuously relieve spasms remain elusive. A larger cohort and longer follow-up study are warranted in the future. Additionally, it would be imperative to fully clarify the mechanism of DN in alleviating muscle spasms.

## Conclusion

Dry needling can relieve varying degrees of hand spasticity instantly in post-stroke patients.

## Data Availability Statement

The raw data supporting the conclusions of this article will be made available by the authors, without undue reservation.

## Ethics Statement

The studies involving human participants were reviewed and approved by the Medical Ethical Review Committee of the Seventh People's Hospital affiliated to Shanghai University of Traditional Chinese Medicine (2018-IRBQYYS). The patients/participants provided their written informed consent to participate in this study.

## Author Contributions

WF, ZZ, KL, WW, and YS equally contributed to the design and implementation of the work. YZ, LJia, QL, and LJin were in charge of data collection. TZ was responsible for data analysis and interpretation. ZZ, KL, and TZ assisted in drafting the manuscript, which was critically revised by WF and finally approved by all the authors.

## Funding

This work was supported by the Shanghai Science and Technology Commission (grant number 18401900300), the National Natural Science Foundation of China (grant number 81873328), and the Shanghai Characteristic Diagnosis and Treatment Technology Improvement Project of Traditional Chinese Medicine [grant number YZ (2018-2020)-ZYJS-04].

## Conflict of Interest

The authors declare that the research was conducted in the absence of any commercial or financial relationships that could be construed as a potential conflict of interest.

## Publisher's Note

All claims expressed in this article are solely those of the authors and do not necessarily represent those of their affiliated organizations, or those of the publisher, the editors and the reviewers. Any product that may be evaluated in this article, or claim that may be made by its manufacturer, is not guaranteed or endorsed by the publisher.

## References

[1] Chen H Shi L Wang N Han Y Lin Y Dai M. Analysis on geographic variations in hospital deaths and endovascular therapy in ischaemic stroke patients: an observational cross-sectional study in China. BMJ Open. (2019) 9:e029079. 10.1136/bmjopen-2019-029079 31239305 PMC6597735

[2] Wang W Jiang B Sun H Ru X Sun D Wang L. Prevalence, incidence, and mortality of stroke in china: results from a nationwide population-based survey of 480 687 adults. Circulation. (2017) 135:759–71. 10.1161/CIRCULATIONAHA.116.025250 28052979

[3] Thibaut A Chatelle C Ziegler E Bruno MA Laureys S Gosseries O. Spasticity after stroke: physiology, assessment and treatment. Brain Injury. (2013) 27:1093–105. 10.3109/02699052.2013.804202 23885710

[4] Wissel J Manack A Brainin M. Toward an epidemiology of poststroke spasticity. Neurology. (2013). 80(3 Suppl 2):S13–9. 10.1212/WNL.0b013e3182762448 23319481

[5] Ghai A Garg N Hooda S Gupta T. Spasticity - pathogenesis, prevention and treatment strategies. Saudi J Anaesth. (2013) 7:453–60. 10.4103/1658-354X.121087 24348300 PMC3858699

[6] Santamato A Micello MF Panza F Fortunato F Baricich A Cisari C. Can botulinum toxin type A injection technique influence the clinical outcome of patients with post-stroke upper limb spasticity? A randomized controlled trial comparing manual needle placement and ultrasound-guided injection techniques. J Neurol Sci. (2014) 347:39–43. 10.1016/j.jns.2014.09.016 25263601

[7] Sun LC Chen R Fu C Chen Y Wu Q Chen R. Efficacy and safety of botulinum toxin type a for limb spasticity after stroke: a meta-analysis of randomized controlled trials. Biomed Res Int. (2019) 2019:8329306. 10.1155/2019/8329306 31080830 PMC6475544

[8] Zheng MX Hua XY Feng JT Li T Lu YC Shen YD. Trial of contralateral seventh cervical nerve transfer for spastic arm paralysis. N Engl J Med. (2018) 378:22–34. 10.1056/NEJMoa1615208 29262271

[9] Kearns G Fernández-De-Las-Peñas C Brismée JM Gan J Doidge J. New perspectives on dry needling following a medical model: are we screening our patients sufficiently? J Man Manip Ther. (2019) 27:172–9. 10.1080/10669817.2019.1567011 30935332 PMC6598537

[10] Tang L Li Y Huang QM Yang Y. Dry needling at myofascial trigger points mitigates chronic post-stroke shoulder spasticity. Neural Regen Res. (2018) 13:673–6. 10.4103/1673-5374.230293 29722319 PMC5950677

[11] Fernández-de-Las-Peñas C Pérez-Bellmunt A Llurda-Almuzara L Plaza-Manzano G De-la-Llave-Rincón AI Navarro-Santana MJ. Is dry needling effective for the management of spasticity, pain, and motor function in post-stroke patients? A systematic review and meta-analysis. Pain Med. (2021). 22:131–41. 10.1093/pm/pnaa392 33338222

[12] Mendigutia-Gómez A Martín-Hernández C Salom-Moreno J. Fernández-de-Las-Peñas C. Effect of dry needling on spasticity, shoulder range of motion, and pressure pain sensitivity in patients with stroke: a crossover study. J Manipul Physiol Therapeut. (2016) 39:348–58. 10.1016/j.jmpt.2016.04.006 27167369

[13] Aho K Harmsen P Hatano S Marquardsen J Smirnov VE Strasser T. Cerebrovascular disease in the community: results of a WHO collaborative study. Bull World Health Organ. (1980) 58:113–30. 6966542 PMC2395897

[14] Liu Z Liu Y Xu H He L Chen Y Fu L. Effect of electroacupuncture on urinary leakage among women with stress urinary incontinence: a randomized clinical trial. Jama. (2017) 317:2493–501. 10.1001/jama.2017.7220 28655016 PMC5815072

[15] Ghannadi S Shariat A Ansari NN Tavakol Z Honarpishe R Dommerholt J. The effect of dry needling on lower limb dysfunction in poststroke survivors. J Stroke Cerebrovasc Dis. (2020) 29:104814. 10.1016/j.jstrokecerebrovasdis.2020.104814 32327366

[16] Salom-Moreno J Sánchez-Mila Z Ortega-Santiago R Palacios-Ceña M Truyol-Domínguez S. Fernández-de-las-Peñas C. Changes in spasticity, widespread pressure pain sensitivity, and baropodometry after the application of dry needling in patients who have had a stroke: a randomized controlled trial. J Manipul Physiol Therapeut. (2014) 37:569–79. 10.1016/j.jmpt.2014.06.003 25199825

[17] Núñez-Cortés R Cruz-Montecinos C Latorre-García R Pérez-Alenda S Torres-Castro R. Effectiveness of dry needling in the management of spasticity in patients post stroke. J Stroke Cerebrovasc Dis. (2020) 29:105236. 10.1016/j.jstrokecerebrovasdis.2020.105236 33066920

[18] Sánchez-Mila Z Salom-Moreno J. Fernández-de-Las-Peñas C. Effects of dry needling on post-stroke spasticity, motor function and stability limits: a randomised clinical trial. Acupunct Med. (2018) 36:358–66. 10.1136/acupmed-2017-011568 29986902

[19] Cuenca Zaldívar JN Calvo S Bravo-Esteban E Oliva Ruiz P Santi-Cano MJ Herrero P. Effectiveness of dry needling for upper extremity spasticity, quality of life and function in subacute phase stroke patients. Acupunct Med. (2021) 39:299–308. 10.1177/0964528420947426 32815384

[20] Ansari NN Naghdi S Fakhari Z Radinmehr H Hasson S. Dry needling for the treatment of poststroke muscle spasticity: a prospective case report. NeuroRehabilitation. (2015) 36:61–5. 10.3233/NRE-141192 25547766

[21] Ghaffari MS Shariat A Honarpishe R Hakakzadeh A Cleland JA Haghighi S. Concurrent effects of dry needling and electrical stimulation in the management of upper extremity hemiparesis. J Acupunct Meridian Stud. (2019) 12:90–4. 10.1016/j.jams.2019.04.004 31026521

[22] Fakhari Z Ansari NN Naghdi S Mansouri K Radinmehr H. A single group, pretest-posttest clinical trial for the effects of dry needling on wrist flexors spasticity after stroke. NeuroRehabilitation. (2017) 40:325–36. 10.3233/NRE-161420 28222554

[23] Lu Z Briley A Zhou P Li S. Are there trigger points in the spastic muscles? Electromyographical evidence of dry needling effects on spastic finger flexors in chronic stroke. Front Neurol. (2020) 11:78. 10.3389/fneur.2020.00078 32153489 PMC7047231

[24] Hernández-Ortíz AR Ponce-Luceño R Sáez-Sánchez C García-Sánchez O. Fernández-de-Las-Peñas C, de-la-Llave-Rincón AI. Changes in muscle tone, function, and pain in the chronic hemiparetic shoulder after dry needling within or outside trigger points in stroke patients: a crossover randomized clinical trial. Pain Med. (2020) 21:2939–47. 10.1093/pm/pnaa132 32488238

